# Epidemiology of primary and revision total knee arthroplasty: analysis of demographics, comorbidities and outcomes from the national inpatient sample

**DOI:** 10.1186/s42836-023-00175-6

**Published:** 2023-04-02

**Authors:** Fong H. Nham, Ishan Patel, Leo Zalikha, Mouhanad M. El-Othmani

**Affiliations:** 1 DMC Orthopaedics & Sports Medicine, 3990 John R Street, Detroit, MI 48201 USA; 2 Department of Orthopaedic Surgery, Columbia University Medical Center, 622 W 168Th Street, New York, NY 10032 USA

**Keywords:** Primary total knee arthroplasty, Revision total knee arthroplasty, Demographics, Comorbidities, Outcomes

## Abstract

**Introduction:**

Primary total knee arthroplasty (TKA) is a preferred treatment for end-stage knee osteoarthritis. In the setting of a failed TKA, revision total knee arthroplasty (rTKA) acts as a salvage procedure and carries a higher risk compared to primary TKA. Given increased interest in postoperative outcomes from these procedures, a thorough understanding of the demographics, comorbidities, and inpatient outcomes is warranted. This study aimed to report the epidemiological data of demographics, comorbidity profiles and outcomes of patients undergoing TKA and rTKA.

**Methods:**

A retrospective review of NIS registry discharge data from 2006 to 2015 third quarter was performed. This study included adults aged 40 and older who underwent TKA or rTKA. A total of 5,901,057 TKA patients and 465,968 rTKA patients were included in this study. Simple descriptive statistics were used to present variables on demographics, medical comorbidities, and postoperative complications.

**Results:**

A total of 5,901,057 TKA and 465,968 rTKA discharges were included in this study, with an average age of 66.30 and 66.56 years, and the major payor being Medicare, accounting for 55.34% and 59.88% of TKA and rTKA cases, respectively. Infection (24.62%) was the most frequent reason for rTKA, and was followed by mechanical complications (18.62%) and dislocation (7.67%). The most common medical comorbidities for both groups were hypertension, obesity, and diabetes. All types of inpatient complications were reported in 22.21% TKA and 28.78% of rTKA cases. Postoperative anemia was the most common complication in both groups (20.34% *vs*. 25.05%).

**Conclusions:**

Our data demonstrated a 41.9% increase in patients receiving TKA and 28.8% increase in rTKA from the years 2006 to 2014. The data showed a 22.21% and a 28.78% “complication” rate with TKA and rTKA, with postoperative anemia being the most common complication. The top 3 medical comorbidities were hypertension, obesity, and diabetes for both groups and with increased focus on perioperative optimization, future analyses into preoperative medical optimization, and improved primary arthroplasty protocol may result in improved postoperative outcomes.

## Introduction

Primary total knee arthroplasty (TKA) is an effective procedure to improve function and provide pain relief in patients with advanced osteoarthritis [1]. Arthritis is projected to impact 78.4 million adults by 2040 [2], with lifetime prevalence of symptomatic knee arthritis estimated at 44.7% [3]. The United States expects an increasing demand for TKA as the 75 million “baby boomer” are predicted to be affected by arthritis at a rate of 50% by 2030 [4].

TKA ranked as the second most common procedure performed in the US in 2018, with a 134% increase from 2005 [5]. While excellent outcomes following TKA have been demonstrated with implant survivorship exceeding 90%, the procedure is not without complications [6]. Revision total knee arthroplasty (rTKA) is a salvage procedure in the setting of a failed TKA, with a current $2.7 billion impact on the US healthcare system [6]. Given the level of complexity, postoperative outcomes following rTKA carries increased risk of complications, including increased hospital length of stay (LOS), operative time, and cost of care [7].

Healthcare systems shifted focus towards minimizing cost while improving quality of delivered care as a continued trend throughout the last decade towards the conceptualization of value-based care. The implementation of systematic perioperative care pathways, such as the Enhanced Recovery After Surgery Society's protocols, demonstrated reduced LOS and increasing home discharge [8]. Despite the reported success in improved postoperative outcomes, perioperative optimization protocols targeting medical and social comorbidities common within arthroplasty patients continue to pose potential for continuous improvement efforts. While previous epidemiological studies highlighted demographics and outcomes for TKA patients, there remains a deficiency of an in-depth, comprehensive analysis of common medical comorbidities among TKA and rTKA recipients.

The aim of this study was to provide a thorough understanding of the comorbidity profile, demographics, and in-hospital outcomes of TKA and rTKA recipients. Furthermore, the indications for rTKA and type of revision performed during the study duration in the United States were reported. Through this report, the authors aimed to provide an in-depth understanding of medical comorbidities and demographics of patients undergoing TKA and rTKA, which could form a critical starting point to design effective optimization protocols for this population.

## Methods

A retrospective analysis of data collected from the National Inpatient Sample (NIS) was performed using discharge data from the years 2006 through the third quarter of 2015. The NIS database comprises a stratified 20% sample of inpatient stays within the United States and contains demographic information, comorbidity profiles, and in-hospital outcomes. Specifically, to rTKA, it also includes types and reasons for revision surgery. The International Classification of Diseases, Ninth Revision, Clinical Modification (ICD-9-CM) was used to search for procedure and diagnosis codes within the NIS. We elected to exclude patients from the fourth quarter of 2015 as an effort to maintain integrity of the methodology and homogeneity of the database, as the NIS had transitioned to the ICD-10 coding system starting in that quarter. Patients were identified with ICD code 81.54 (total knee replacement) or 81.55 (revision of total knee replacement). Reasons for revision were also included with the use of ICD codes: 996.42 (dislocation/instability), 996.41 (mechanical loosening), 996.66 (infection), 996.43 (implant failure), 996.45 (periprosthetic osteolysis), 996.44 (periprosthetic fracture), 996.46 (bearing surface wear), 996.47 (other mechanical problems) and 996.49 (other mechanical complications). We chose to combine "other mechanical problems" and "other mechanical complications" into one category in the results. The type of revision was also included with ICD codes: 00.80 (all components), 80.06 (arthrotomy for removal of prosthesis), 00.84 (tibial insert), 00.81 (tibial component), 00.83 (patellar), 00.82 (femoral component), and 81.55 (others, not otherwise specified). All patients aged 40 years or older were included in this study to provide a representative national sample of typical patients undergoing the procedures.

Demographic data collected included calendar year, age, race, sex, primary payor, elective *vs.* non-elective admission, as well as region and bed size of hospital. In-hospital outcome data harvested included average cost, LOS, and complications during the inpatient stay as reported in the database. This dataset includes cardiac complications, respiratory complications, peripheral vascular disease (PVD) complications, hematoma/seroma, wound dehiscence, postoperative infection, gastrointestinal complication, genitourinary complication, deep vein thrombosis, pulmonary embolism or postoperative anemia, which collectively are referred to as "any complications". A comorbidity profile using the Elixhauser comorbidity index, which is frequently used in database studies to evaluate patient comorbidities, is presented to highlight the common medical comorbidities with potential impact on postoperative outcomes [9].

## Results

### Demographic data

A total of 5,901,057 TKA and 465,968 rTKA discharges were included in this study. The average age in the TKA and rTKA groups was 66.30 and 66.56 years, respectively. The major payor was Medicare at 55.34% and 59.88% of TKA and rTKA cases, respectively. Non-Hispanic whites were the majority race receiving TKA and rTKA discharges at 71.25% and 70.96% of discharges, respectively.

**Fig. 1:**
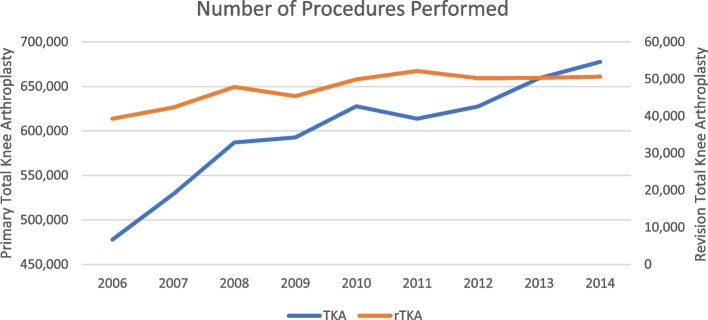
Number of primary and revision total knee arthroplasties performed by year and not including data from the last quarter of 2015

Overall, there was an increase of 41.81% and 28.79% in TKA and rTKA procedures from the year 2006 to 2014. The decrease in 2015 can be attributed to our methodology, which did not include the 4th quarter data. Figure 1 is graphs depicting the number of TKA and rTKA procedures performed from the years 2006 to 2014. Table 1 is a summary of the demographic variables of the study population.

**Table 1:** Comprehensive list of demographic data collected from the NIS database of patients undergoing TKA and rTKA from 2006 to the third quarter of 2015

	**TKA (*n* = 5,901,057)**	**rTKA (*n* = 465,968)**
Age of Patient (Years)		
Mean (Standard Error)	66.30 (0.04)	66.56 (0.06)
Biological Sex of Patient		
Male	2,197,685 (37.24%)	207,630 (44.56%)
Female	3,703,371 (62.76%)	258,338 (55.44%)
Expected Primary Payor		
Medicare	3,265,481 (55.34%)	279,009 (59.88%)
Private	2,226,286 (37.73%)	140,970 (30.25%)
Self-Pay	26,031 (0.44%)	2,474 (0.53%)
No Charge	4,680 (0.08%)	357 (0.08%)
Other Insurance	203,329 (3.45%)	25,254 (5.42%)
Race of Patient		
Non-Hispanic White	4,204,427 (71.25%)	330,671 (70.96%)
Non-Hispanic Black	378,244 (6.41%)	38,451 (8.25%)
Hispanic	274,034 (4.64%)	19,462 (4.18%)
Calendar Year of Discharge		
2006	477,858 (8.10%)	39,303 (8.44%)
2007	529,082 (8.97%)	42,324 (9.08%)
2008	586,937 (9.95%)	47,870 (10.27%)
2009	592,657 (10.07%)	45,358 (9.73%)
2010	627,691 (10.64%)	49,894 (10.71%)
2011	613,742 (10.40%)	52,149 (11.19%)
2012	627,534 (10.63%)	50,235 (10.78%)
2013	659,295 (11.17%)	50,315 (10.80%)
2014	677,656 (11.48%)	50,620 (10.86%)
2015	508,604 (8.62%)	37,900 (8.13%)
Bedsize of Hospital		
Small	1,168,666 (19.80%)	81,509 (17.49%)
Medium	1,561,211 (26.46%)	119,456 (25.64%)
Large	3,150,403 (53.39%)	262,634 (56.36%)
Unknown	20,777 (0.35%)	2,369 (0.51%)
Location/Teaching Status of Hospital		
Rural	695,816 (11.79%)	42,418 (9.10%)
Urban Nonteaching	2,577,382 (43.68%)	183,223 (39.32%)
Urban Teaching	2,607,082 (44.18%)	237,958 (51.07%)
Unknown	20,777 (0.35%)	2,369 (0.51%)
Region of Hospital		
Northeast	988,218 (16.75%)	80,848 (17.35%)
Midwest	1,617,066 (27.40%)	117,620 (25.24%)
South	2,156,781 (36.55%)	177,236 (38.04%)
West	1,138,992 (19.30%)	90,263 (19.37%)
Elective Admission		
Non-Elective	*n*/a	80,947 (17.37%)
Elective	*n*/a	383,949 (82.40%)
Unknown	*n*/a	1,072 (0.23%)

### Elixhauser comorbidity profile

Hypertension was the most common comorbidity in both TKA and rTKA groups, accounting for 67.74% and 65.04%. The five most prevalent comorbidities in the TKA group were hypertension (67.74%), obesity (21.73%), uncomplicated diabetes (19.83%), hypothyroidism (15.57%) and chronic pulmonary disease (14.79%). The five most prevalent comorbidities in the rTKA group included hypertension (65.04%), obesity (22.98%), uncomplicated diabetes (19.82%), chronic pulmonary disease (16.54%) and deficiency anemia (15.67%). The full Elixhauser comorbidity profile for both TKA and rTKA is summarized in Table 2.

**Table 2:** Elixhauser comorbidity profile collected from the NIS database of patients undergoing TKA and rTKA from 2006 to the third quarter of 2015

	**TKA**	**rTKA**
	Discharges (*n* = 5,901,057)	Discharges (*n* = 465,968)
Hypertension	3,997,142 (67.74%)	303,063 (65.04%)
Obesity	1,282,015 (21.73%)	139,138 (22.98%)
Uncomplicated Diabetes	1,170,292 (19.83%)	92,345 (19.82%)
Hypothyroidism	918,912 (15.57%)	69,510 (14.92%)
Chronic Pulmonary Disease	872,937 (14.79%)	77,083 (16.54%)
Depression	746,350 (12.65%)	67,245 (14.43%)
Deficiency Anemias	740,986 (12.56%)	73,030 (15.67%)
Renal Failure	223,995 (3.97%)	30,362 (6.52%)
Rheumatoid Arthritis/Collagen Vascular Disease	227,961 (3.86%)	29,431 (6.32%)
Other Neurological Disorders	227,335 (3.85%)	23,901 (5.13%)
Valvular Disease	210,353 (3.57%)	21,333 (4.58%)
Congestive Heart Failure	146,451 (2.48%)	23,732 (5.09%)
Psychoses	121,032 (2.05%)	13,002 (2.79%)
Peripheral Vascular Disorders	117,924 (2.00%)	12,417 (2.67%)
Coagulopathy	109,935 (1.86%)	13,889 (2.97%)
Complicated Diabetes	102,091 (1.73%)	11,439 (2.46%)
Chronic Blood Loss Anemias	92,020 (1.56%)	8,420 (1.81%)
Pulmonary Circulation Disorders	56,952 (0.97%)	6,222 (1.34%)
Liver Disease	52,308 (0.89%)	7,274 (1.56%)
Alcohol Abuse	49,504 (0.84%)	5,610 (1.20%)
Drug Abuse	26,544 (0.45%)	4,719 (1.01%)
Solid Tumor without Metastasis	24,025 (0.41%)	2,376 (0.51%)
Paralysis	14,952 (0.25%)	1,909 (0.41%)
Lymphoma	13,509 (0.23%)	1,956 (0.42%)
Weight Loss	13,221 (0.22%)	6,132 (1.32%)
Metastatic Cancer	3,979 (0.07%)	871 (0.19%)
Acquired Immune Deficiency Syndrome (AIDS)	1,089 (0.02%)	185 (0.04%)
Peptic Ulcer Disease Excluding Bleeding	1,199 (0.02%)	94 (0.02%)

### In-hospital outcomes

Any complication occurred after TKA in 22.21% of cases and after rTKA in 28.78%. The most common complication for both TKA and rTKA was postoperative anemia, making up 20.34% and 25.05% respectively. In terms of cost, TKA had a total cost of $49,789 with an average LOS of 3.22 days while rTKA incurred a total cost of $71,872, with an average LOS of 4.44 days. Table 3 summarizes all in-hospital outcomes for both TKA and rTKA.

**Table 3:** In-hospital outcomes collected from the NIS database of patients undergoing TKA and rTKA from 2006 to the third quarter of 2015

	**TKA**	**rTKA**
	Discharges (*n* = 5,901,057)	Discharges (*n* = 465,968)
Any Complication	1,310,652 (22.21%)	134,100 (28.78%)
Surgical Complications		
Postoperative Anemia	1,200,026 (20.34%)	116,702 (25.05%)
Hematoma/Seroma	33,447 (0.57%)	7,772 (1.67%)
Postoperative Infection	8,401 (0.14%)	3,672 (0.79%)
Peripheral Vascular Disease (PVD) Complication	8,045 (0.14%)	688 (0.15%)
Wound Dehiscence	5,777 (0.10%)	6,618 (1.42%)
Medical Complications		
Cardiac Complication	36,702 (0.62%)	2,786 (0.60%)
Genitourinary (GU) Complication	29,033 (0.49%)	1,890 (0.41%)
Pulmonary Embolism (PE)	24,466 (0.41%)	1,654 (0.36%)
Deep Vein Thrombosis (DVT)	22,677 (0.38%)	2,869 (0.62%)
Respiratory Complication	10,39 (0.18%)	1,606 (0.34%)
Died During Hospitalization	3,978 (0.07%)	1,305 (0.28%)
Length of Stay (LOS, Standard error)	3.22 (0.01)	4.44 (0.03)
Total Charges ($, Standard error)	$49,789 ($418)	$71,872 ($832)

### Reasons/types of rTKA

Total joint infection (24.62%) was the most common reason for rTKA. This was followed by mechanical loosening (19.74%), and other mechanical complications (18.62%). With regard to type of revision, the majority of patients underwent revision of all components (37.23%). The tibial component (20.75%) was revised more often than the insert or femoral component (15.68% and 15.13%, respectively). The reason and type of rTKA are summarized in Tables 4, 5, respectively.

**Table 4:** Types of rTKA collected from the NIS database from 2006 to the third quarter of 2015

	**rTKA**
	Discharges (*n* = 46,5968)
All Components	173,500 (37.23%)
Tibial Component	96,694 (20.75%)
Tibial Insert	73,073 (15.68%)
Femoral Component	70,509 (15.13%)
Arthrotomy for Removal of Prosthesis	61,474 (13.19%)
Other, Not Otherwise Specified	27,883 (5.98%)

**Table 5:** Indications for rTKA collected from the NIS database from 2006 to the third quarter of 2015

	**rTKA**
	Discharges (*n* = 46,5968)
Infection	114,719 (24.62%)
Other Mechanical Problems	86,778 (18.62%)
Dislocation/Instability	35,731 (7.67%)
Implant	24,765 (5.31%)
Bearing Surface Wear	17,341 (3.72%)
Periprosthetic Osteolysis	12,483 (2.68%)
Periprosthetic Fracture	7,388 (1.59%)
Mechanical Loosening	1,987 (0.43%)

## Discussion

TKA volume in the US is projected to increase 153% from 2012 to 2050 [2], with an expected increase in rTKA. Our study demonstrated an increase of 41.81% and 28.79% in TKA and rTKA cohorts from the year 2006 to 2014. The increase in TKA volume is likely related to an increasing demand and expanding indications for TKA, possibly due to increasing age, diagnostic modalities identifying end-stage osteoarthritis, and improvement in implant manufacturing [10]. As primary and revision procedure volume increases, there has been a shift towards efficient resource utilization and elevating quality of care delivery. In our study, TKA was found to incur an average total cost of $49,789 per procedure, with an average LOS of 3.22 days, while rTKA had a total inpatient cost of $71,872 per procedure, with an average LOS of 4.44 days. The projected increase in rTKA is expected to pose a heavy financial burden on the healthcare system [6]. Improved perioperative optimization of medical and social comorbidities has been shown to improve outcomes and decrease failure of TKA, potentially serving as an opportunity to decrease the revision burden and lead to health care savings [11]. While perioperative optimization has been an apparent effort in TKA, similar efforts should be made on rTKA recipients given the complexity and risk associated with the procedure. Therefore, we aimed to provide a thorough understanding of the demographics and comorbidities of TKA and rTKA recipients.

Despite the widespread success of TKA in treating end-stage osteoarthritis, failed TKA and the need for subsequent revisions present a significant burden to the healthcare system and patients' quality of life. This study demonstrated a 28.79% increase between 2006 to 2014 in rTKA incidence, with infection remaining the most common reason for rTKA and accounting for 24.62% of all revisions. This was similar to an epidemiological study from Bozic et al*.* between 2005–2006, which identified infection as the most common cause of revision, accounting for 25.2% of all revisions [12]. Their study also had an average LOS of 5.1 days and an average total inpatient cost of $49,360, resulting in a substantially longer LOS (4.44 for rTKA) as compared to findings, which might be attributed to more recent data in this study. Kamath et al. reported several implicating factors for infections as a cause of revision, with medical comorbidities as a risk factor, suggesting that the risk could be reduced through appropriate patient screening, medical interventions, and risk assessment to optimize patient in the perioperative setting [13]. As to medical comorbidities and demographics, their study also implicated that younger age, obesity, and socioeconomic status were also culpable for increased rates of infection. Preoperative screening and appropriate medical interventions with appropriate consultation may decrease the overall complications following rTKA in the setting of infection.

Demographic variables of this study were similar to previously reported age, gender distribution, hospital size, and location/teaching status of hospital [14]. Our study noted the average age of TKA and rTKA patients was 66.30 and 66.56, respectively, with females accounting for 62.76% of TKA and 55.44% of rTKA. With regard to race, non-Hispanic whites accounted for 71.25% of TKA recipients and 70.96% for rTKA. TKA and rTKA were performed mainly in large hospitals 53.59% and 56.36% of the time, respectively. TKA and rTKA were performed at urban teaching hospitals with a rate of 44.18% and 51.07%, respectively. Case complexity may explain the discrepancy in the frequency, but there are likely reimbursement and financial incentivization guiding shifting patterns [15]. Previous studies have described the unequal and inadequate reimbursement systems for compensation for rTKA compared to TKA [16, 17]. Non-teaching community centers may be incentivized indirectly to “cherry pick” primary TKA and “lemon dropping” the complex and inadequately compensated revision procedures, referring patients to urban teaching centers [16].

This study reported on the comorbidities that constitute the Elixhauser comorbidity index, which has been proven to be a superior tool for outcome prediction at population level through extensive utilization in epidemiologic studies [9, 18, 19]. Thorough analysis and understanding of medical comorbidities may serve to guide development of risk stratification models, improve preoperative optimization care protocols, and potentially inform adequate reimbursement models. There exists a deficiency in the impact of major comorbidities and the respective interaction on postoperative outcomes within the arthroplasty literature. The comorbidities most common commodities in this study for both TKA and rTKA were hypertension, obesity, uncomplicated diabetes, hypothyroidism, chronic pulmonary disease, depression, deficiency anemia, and fluid/electrolyte disorders.

Obesity has been extensively studied as a comorbidity in TKA [20]. In this study, it was found to be the second most common comorbidity in both TKA and rTKA. Hijazi et al. reported a correlation between increasing BMI and postoperative outcomes, including increased LOS, total cost, and blood loss, leading to postoperative complications [20]. A systematic review by McElroy et al*.* demonstrated morbid obesity was associated with higher complication rates, lower implant survivorship, and lower postoperative function scores, suggesting a BMI of 40 as a cutoff to guide perioperative optimization [21]. Future research assessing the rates of obesity among TKA and rTKA recipients in more recent years can provide a better understanding of trends in offering the procedure to this population, given that the large body of evidence indicated a higher risk of complications among obese patients.

Diabetes, as a preoperative comorbidity, has been shown to affect post-TKA outcomes. Na et al*.* demonstrated a significantly higher readmission risk with uncontrolled diabetes in Medicare recipients. The authors suggested diabetes and associated systemic complications should be included in reimbursement models [22]. The effect of hypertension effect on cardiac complications are debated in the literature. A systematic review by Elsiwy et al. identified four independent studies on the effect of hypertension on primary total joint arthroplasty outcomes, with two articles reporting no effect and two suggesting a positive correlation. The authors suggested that a positive history of cardiac disease bears the strongest association with cardiac complications postoperatively [23]. Optimization of these medical comorbidities may reduce the burden of health care system by improving postoperative outcomes, including LOS, home discharge, re-admission, and function. A study by Bernstein et al*.* found that optimization of medical comorbidities by screening demonstrated patients had shorter average LOS and lower costs following total joint arthroplasty [5].

Recently, machine learning algorithms have been gaining popularity in the prediction of postoperative outcomes after TKA [24, 25]. Comorbidities can be incorporated into such algorithms to develop accurate models of patient risk stratification. When models become more readily available and validated with predictable accuracy, adequate reimbursement models with patient-specific payment models can be systematically implemented. Ramkumar et al*.* utilized an artificial neural network trained with NIS patient variables to predict LOS, total cost, and discharge disposition [25]. The authors concluded with an acceptable model to be applied towards tiered reimbursement by increasing complexity. These patient-specific payment models can potentially influence physician's “lemon-dropping” by adequately compensating for case complexity.

Similar to other large registry cross-sectional studies, this study has several limitations. While the NIS supplies vast data on healthcare resource utilization at a population derived level, there are errors inherent to suboptimal coding and manual entry of data [26]. Although there is an inherent weakness, the NIS database has been validated as a reliable source of comorbidity and complication data, proved to be an excellent tool for observational population epidemiological studies [12]. Additionally, NIS data are limited to inpatient data during the in-hospital period and does not account for long-term postoperative outcomes and follow up in the global period. Future studies identifying long-term postoperative outcomes can provide utility in perioperative optimization efforts.

Despite the inherent database limitations, this study presented the largest available population level report stratifying medical comorbidities of TKA and rTKA patients. The duration of the study and volume of data provided an in-depth inquiry of demographics, medical comorbidities, postoperative clinical and economic outcomes following TKA and rTKA. The comprehensive information provided allows for a generalizable understanding of perioperative comorbidities to facilitate future studies to further understand the demographics and medical comorbidities among TKA and rTKA recipients. This study aimed to achieve a baseline understanding of common perioperative conditions that have a potential impact on postoperative outcomes.

## Conclusion

In conclusion, this study analyzed the epidemiological and demographic characteristics of TKA and rTKA recipients between 2006–2015. There was an increase of 41.9% and 28.8% in TKA and rTKA procedures, respectively. This study demonstrated anemia had a 22.21% and a 28.78% in-patient rate of occurrence with TKA and rTKA, and was the most commonly reported complication. As focus is increasingly placed on value-based care with optimization of care delivery and minimization of medical waste, a thorough understanding of patient comorbidities is critical to developing care protocol pathways to systematically improve outcomes. This study highlights important areas for future studies on targeted interventions for various patients undergoing TKA or rTKA.

## Data Availability

The datasets generated and/or analysed during the current study are available in the National Inpatient Sample Repository, https://www.hcup-us.ahrq.gov/db/nation/nis/nisdbdocumentation.jsp.
